# PLANNING FOR OLD AGE BY GRADUATES OF A MASTER’S GERONTOLOGY PROGRAM IN ISRAEL

**DOI:** 10.1093/geroni/igad104.1486

**Published:** 2023-12-21

**Authors:** Ella Cohn-Schwartz, Doron Sagi, Lian Meiry

**Affiliations:** Ben Gurion University, Haifa, HaZafon, Israel; College of Law and Business, Ramat Gan, Israel (CLB), Maitar, HaDarom, Israel; Faculty of Health Sciences at Ben-Gurion University of the Negev, Beersheba, HaDarom, Israel

## Abstract

The master’s programs in gerontology in Israel were founded in 1999 in response to the challenges brought by extended life courses and the accompanying accelerated growth of welfare and health services for the aged. Since its beginning, thousands of students graduated from the programs and occupy central positions in the gerontological field. However, no empirical data exists on the extent of the involvement by graduates in employment in the field of aging. The current study aims to provide a first-time assessment of the experiences of graduates in Israel following their studies, as well as examine the level of preparedness to old age among graduates, since their work and study of older adults might make their own future old age more salient. We collected data using online surveys from 197 graduates of the gerontology programs. Participants were asked about their work experience in the gerontology field and about the extent to which they prepare for old age. The findings showed that most graduates (80%) currently work in the field of aging, and are overall pleased with the jobs. However, most of the graduated (68%) also reported difficulties in finding employment. Logistic regressions showed that those who currently work in aging and who graduated before less years were also less likely to make preparations for needing nursing care in old age and talking with relatives about their death. Thus, working and studying about aging could be related to being less likely to make preparations for old age.

